# Modeling recent gun purchases: A social epidemiology of the pandemic arms race

**DOI:** 10.1016/j.pmedr.2021.101634

**Published:** 2021-11-14

**Authors:** Terrence D. Hill, Ming Wen, Christopher G. Ellison, Guangzhen Wu, Benjamin Dowd-Arrow, Dejun Su

**Affiliations:** a Department of Sociology, The University of Texas at San Antonio, One UTSA Circle, San Antonio, TX 78249, USA; b Department of Sociology, The University of Utah, 390 1530 E #301, Salt Lake City, UT 84112, USA; c Public Health Program, Florida State University, 526 Bellamy Building, Tallahassee, FL 32306, USA; d Department of Health Promotion, The University of Nebraska Medical Center, 984340 Nebraska Medical Center, Omaha, NE 68198, USA

**Keywords:** Gun ownership, Pandemic, Social epidemiology

## Abstract

In this paper, we document the social patterning of recent gun purchases to advance a contemporary social epidemiology of the pandemic arms race. We employ cross-sectional survey data from the 2020 *Health, Ethnicity and Pandemic Study*, which included a national sample of 2,709 community-dwelling adults living in the United States. We use binary logistic regression to model recent pandemic gun purchases as a function of age, sex, race/ethnicity, nativity status, region of residence, marital status, number of children, education, household income, pandemic job change, religious service attendance, pandemic religion change, and political party. Overall, 6% of the sample reported purchasing a new gun during the pandemic. Multivariate regression results suggest that pandemic gun purchasers tend to be male, younger, US-born, less educated, recently unemployed, experiencing changes in their religious beliefs, Republicans, and residents of southern states. To our knowledge, we are among the first to formally document a new population of pandemic gun owners that is characterized by youth, US-nativity, and religious volatility. Our analyses underscore the need for public health initiatives designed to enhance gun-related safety during pandemics, including, for example, addressing underlying motivations for recent gun purchases and improving access to training programs.

## Highlights

• We consider the social patterning of recent pandemic gun purchases.
• Overall, 6% of the sample reported purchasing a new gun during the pandemic.
• Pandemic gun purchasers tend to be male, younger, US-born, and living in the south.
• Pandemic gun purchasers tend to be less educated and recently unemployed.
• Pandemic gun purchasers tend to be Republicans and experiencing religious change.

## 1 Introduction

Americans own more guns per capita than any other country in the world. While the United States (US) ranks first with over 120 firearms for every 100 residents, war-torn Yemen ranks second with nearly 60 firearms for every 100 residents (Karp, 2018). There are now growing concerns in public health about how gun sales have skyrocketed in the US during the coronavirus (COVID-19) pandemic (Caputi et al., 2020, Kravitz-Wirtz et al., 2021, Lyons et al., 2021, McCourt, 2021). In 2020, gun sales increased 64% from 2019, and, during the first quarter of 2021, gun sales increased 18% from the first quarter of 2020 (Tavernise, 2021). According to journalistic accounts, Americans are in the midst of a pandemic “arms race with themselves” (Tavernise, 2021).

It is well established that guns are probabilistic threats to public health in the US. For example, studies show that owning a gun or having a gun in the home increases the risk of gun-related suicide and homicide for men, women, and children (Gramlich, 2019, Miller et al., 2002, Miller et al., 2007, Van Kesteren, 2014). In fact, guns are associated with thousands of preventable injuries and premature deaths each year (Fleegler et al., 2013, Fowler et al., 2015, Gani et al., 2017). It should come as no surprise, given the rise in gun sales, that gun-related deaths due to homicides and unintentional shootings have also surged during the pandemic (Bates, 2021, Sutherland et al., 2021). The national health care costs associated with firearm-related injuries are now estimated into the billions annually (Gani et al., 2017, Spitzer et al., 2017).

With all of this in mind, it is perhaps more important than ever to be focused on the social epidemiology of gun ownership. A long tradition of research has identified a number of robust sociodemographic correlates of individual gun ownership among US adults, and many of these social patterns have been consistently observed across studies and over time. For example, we know that gun owners tend to be male, white, older, less educated, more affluent, married with children, religiously conservative, Republican, and residents of southern states and more rural areas (Azrael et al., 2017, Dowd-Arrow et al., 2019, Ellison, 1991, Ellison et al., 2021, Goss, 2017, Hepburn et al., 2007, Kelley and Ellison, 2021, Legault, 2008, Mencken and Froese, 2019, O’Connor and Lizotte, 1978, Parker et al., 2017, Smith et al., 2019, Vegter and Kelley, 2020).

In this paper, we build on this pre-pandemic gun scholarship by documenting the social patterning of recent gun purchases to advance a contemporary social epidemiology of the pandemic arms race. More specifically, we test whether the odds of purchasing a gun during the pandemic vary according to age, sex, race/ethnicity, nativity status, region of residence, marital status, number of children, education, household income, pandemic job change, religious service attendance, pandemic religion change, and political party. Our analyses are potentially important because the population of first-time gun owners has increased considerably during the pandemic (Arnold, 2020, Diaz, 2021, Tavernise, 2021). Although we anticipate a great deal of continuity in the social patterning of recent pandemic gun purchases, we also expect new patterns to emerge as the population of gun owners changes over time. Our analyses may potentially inform the development of public health initiatives designed to enhance gun-related safety during pandemics.

## 2 Materials and methods

### 2.1 Data

We employ data from the 2020 *Health, Ethnicity and Pandemic Study* (HEAP) (Matthews et al., 2021). HEAP is based on a national sample of 2,709 community-dwelling adults aged 18 and over. Respondents were primarily sampled from the National Opinion Research Center’s (NORC) *AmeriSpeak Panel* (National Opinion Research Center, 2021), a national panel survey designed to be representative of US households. Participants were selected from the *AmeriSpeak* panel using 48 sampling strata, including age, race, education, and gender. Asian Americans were oversampled from Dynata’s nonprobability online opt-in panel (Elections Canada, 2021). Sampled respondents were invited to complete the online survey in English or Spanish during the month of October 2020. The data collection process yielded a weighted American Association for Public Opinion Research (AAPOR) Response Rate 3 of 21%. The average web-based survey lasted approximately 12 min. The NORC Institutional Review Board reviewed and approved the survey (IRB #20.10.43). Informed consent was obtained from all participants.

### 2.2 Variables

*Pandemic gun purchases* were assessed using a single item. Respondents were asked: “Have you purchased a gun during the pandemic?” All respondents were informed that the survey was focused on the “COVID-19 pandemic.” The original response categories for this item were (1) yes and (0) no.

Sociodemographic variables include *age* (dummy variables for 18–24, 25–34, 35–44, and 45–69, with 60 and older serving as the reference), *sex* (male = 1; female = 0), *race/ethnicity* (dummy variables for non-Hispanic black, Latino, Asian, and other races and ethnicities, with non-Hispanic white serving as the reference); *nativity status* (1 = US-born; 0 = otherwise), *region of residence* (dummy variables for Northeast, Midwest, and West, with South serving as the reference), *marital status* (1 = married; 0 = otherwise), *number of children* (continuous count, top-coded at 5), *educational attainment* (dummy variables for high school/GED diploma, some college/associate degree, bachelor’s degree, and graduate degree, with less than high school serving as the reference), *annual household income* (1 = $5,000 to 18 = $200,000 or above), *pandemic job change* (dummy variables for being continuously employed during the pandemic, being continuously unemployed, and gaining employment, with losing employment during the pandemic serving as the reference), *religious attendance* (dummy variables for sporadic, monthly, and weekly attendance, with never attending religious services being the reference), *pandemic religion change* (dummy variables for no change in one’s religious beliefs during the pandemic, becoming more religious, and becoming less religious, with not being religious serving as the reference), and *political party* (dummy variables for Democrat and Independent/none, with Republican serving as the reference).

### 2.3 Analysis

Our analyses begin with sample descriptive statistics for all study variables, including variable ranges, percentages, means, and standard deviations (Table 1). We then use binary logistic regression to model pandemic gun purchases as a function of sociodemographic variables (Table 2). We present five regression models with odds ratios and 95% confidence intervals. Model 1 regresses pandemic gun purchases on age, sex, race/ethnicity, nativity status, and region of residence. Model 2 adds family characteristics, including marital status and the number of children. Model 3 includes indicators of socioeconomic status, including education, household income, and pandemic job change. Model 4 introduces the religion variables, including religious attendance and pandemic religion change. Finally, Model 5, which adds political party, represents our full model. All analyses were conducted in STATA 15.

**Table 1:** Sample Descriptive Statistics.

	*Percentage/Mean (SD)*	*Range*	*Observation*
Pandemic Gun Purchase	6.18%	[0, 1]	2,686
Age			
18 to 24 years	11.41%	[0, 1]	2,709
25 to 34 years	23.81%	[0, 1]	2,709
35 to 44 years	18.24%	[0, 1]	2,709
45 to 59 years	21.15%	[0, 1]	2,709
≥ 60 years	25.40%	[0, 1]	2,709
Male	47.84%	[0, 1]	2,709
Race and Ethnicity			2,706
White	18.99%	[0, 1]	
Black	21.80%	[0, 1]	
Latino	19.66%	[0, 1]	
Asian	36.10%	[0, 1]	
Others	3.44%	[0, 1]	
US-Born	75.76%	[0, 1]	2,698
South Region	33.44%	[0, 1]	2,709
Marital Status			2,709
Married	45.77%	[0, 1]	
Widowed	2.95%	[0, 1]	
Divorced	7.72%	[0, 1]	
Separated	4.25%	[0, 1]	
Never married	31.78%	[0, 1]	
Living with partner	7.53%	[0, 1]	
Number of Children (top-coded at 5)	0.672 (1.17)	[0, 5]	2,709
Education			2,709
Less than high school	5.68%	[0, 1]	
High school or GED diploma	15.84%	[0, 1]	
Some college or associate degree	39.61%	[0, 1]	
Bachelor’s degree	21.56%	[0, 1]	
Graduate degree	17.31%	[0, 1]	
Household Income	9.82 (4.50)	[1, 18]	2,709
Pandemic Job Change			2,185
Lost employment	10.30%	[0, 1]	
Continuously employed	69.75%	[0, 1]	
Continuously unemployed	17.21%	[0, 1]	
Gained employment	2.75%	[0, 1]	
Religious Attendance			2,703
Never	29.67%	[0, 1]	
Seldom	36.77%	[0, 1]	
Monthly	7.47%	[0, 1]	
Weekly	26.08%	[0, 1]	
Pandemic Religion Change			2,692
No change	61.44%	[0, 1]	
More religious	12.96%	[0, 1]	
Less religious	7.06%	[0, 1]	
Not religious	18.54%	[0, 1]	
Political Party			2,705
Democrat	59.52%	[0, 1]	
Independent/none	16.56%	[0, 1]	
Republican	23.92%	[0, 1]

**Table 2:** Logistic Regression of Gun Purchase during the COVID-19 Pandemic

	*Model 1*		*Model 2*		*Model 3*		*Model 4*		*Model 5*	
	*(n = 2,673)*		*(n = 2,673)*		*(n = 2,158)*		*(n = 2,158)*		*(n = 2,158)*	
Age (Ref = ≥ 60)										
18 to 24 years	3.13***	(1.62 6.02)	3.23***	(1.62 6.47)	2.92*	(1.25 6.86)	2.78*	(1.18 6.57)	2.80*	(1.18 6.63)
25 to 34 years	3.49***	(2.00 6.08)	3.41***	(1.92 6.08)	3.08**	(1.49 6.37)	3.01**	(1.44 6.28)	2.89**	(1.38 6.06)
35 to 44 years	3.49***	(1.94 6.27)	3.21***	(1.76 5.87)	2.65*	(1.25 5.63)	2.81**	(1.31 6.00)	2.61*	(1.21 5.63)
45 to 59 years	2.38**	(1.31 4.31)	2.32**	(1.28 4.21)	2.07+	(0.99 4.36)	2.12*	(1.00 4.48)	2.01+	(0.95 4.27)
Male	1.96***	(1.40 2.73)	1.97***	(1.41 2.76)	1.75**	(1.23 2.51)	1.86***	(1.29 2.68)	1.72**	(1.19 2.50)
Race/ethnicity (Ref = White)										
Black	1.25	(0.78 1.99)	1.29	(0.80 2.07)	1.18	(0.70 1.99)	1.00	(0.58 1.71)	1.74+	(0.98 3.11)
Latino	1.04	(0.63 1.72)	1.04	(0.63 1.72)	1.03	(0.60 1.77)	0.93	(0.53 1.61)	1.24	(0.70 2.19)
Asian	0.77	(0.46 1.29)	0.79	(0.47 1.33)	1.04	(0.58 1.85)	0.97	(0.54 1.75)	1.27	(0.69 2.32)
Other	1.03	(0.41 2.57)	1.02	(0.41 2.56)	1.12	(0.43 2.89)	1.03	(0.39 2.73)	1.27	(0.47 3.42)
US-born	4.07***	(2.13 7.77)	4.14***	(2.16 7.92)	3.83***	(1.98 7.42)	4.00***	(2.06 7.78)	4.30***	(2.20 8.41)
Region (Ref = South)										
Northeast	0.52*	(0.29 0.92)	0.53*	(0.29 0.95)	0.54*	(0.30 0.98)	0.52*	(0.28 0.95)	0.55+	(0.30 1.01)
Midwest	0.73	(0.47 1.15)	0.73	(0.47 1.14)	0.67+	(0.42 1.08)	0.65+	(0.40 1.06)	0.67	(0.41 1.09)
West	0.68+	(0.45 1.02)	0.68+	(0.45 1.02)	0.56*	(0.36 0.88)	0.55**	(0.35 0.86)	0.55*	(0.35 0.88)
Number of Children (top-coded at 5)			1.19	(0.83 1.71)	1.24	(0.83 1.85)	1.18	(0.78 1.78)	1.14	(0.76 1.73)
Education (Ref = < High School)			1.09	(0.97 1.23)	1.08	(0.95 1.22)	1.04	(0.91 1.19)	1.03	(0.90 1.18)
High school or GED diploma					0.82	(0.40 1.69)	0.80	(0.39 1.66)	0.80	(0.38 1.68)
Some college or associate degree					0.75	(0.38 1.48)	0.79	(0.40 1.56)	0.82	(0.41 1.64)
Bachelor's degree					0.57	(0.25 1.28)	0.57	(0.25 1.29)	0.61	(0.27 1.39)
Graduate degree					0.36*	(0.15 0.89)	0.36*	(0.15 0.90)	0.39*	(0.16 0.98)
Household Income					1.01	(0.97 1.06)	1.02	(0.97 1.06)	1.01	(0.96 1.06)
Pandemic Job change (Ref = Lost Employment)										
Continuously Employed					0.93	(0.56 1.56)	0.97	(0.58 1.65)	0.96	(0.56 1.65)
Continuously Unemployed					0.47*	(0.22 0.98)	0.46*	(0.22 0.98)	0.44*	(0.20 0.95)
Gained Employment					0.74	(0.26 2.12)	0.60	(0.21 1.78)	0.55	(0.18 1.68)
Religious Service Attendance (Ref = Never)										
Seldom							0.79	(0.49 1.28)	0.77	(0.47 1.25)
Monthly							0.95	(0.47 1.92)	0.87	(0.42 1.78)
Weekly							1.13	(0.68 1.87)	1.00	(0.59 1.68)
Pandemic Religion Change (Ref = Not Religious)										
No change							1.49	(0.80 2.79)	1.27	(0.67 2.41)
More religious							2.90**	(1.39 6.05)	2.30*	(1.09 4.85)
Less religious							3.79***	(1.75 8.22)	3.39**	(1.55 7.42)
Political Party (Ref = Republican)										
Democrat									0.32***	(0.21 0.49)
Independent/None									0.50**	(0.30 0.84)

*Notes*: Shown are odds ratios and 95% confidence intervals (in parentheses). Two-tailed tests: * p < 0.05; ** p < 0.01; *** p < 0.001.

## 3 Results

### 3.1 Descriptive analyses

According to Table 1, 6% of respondents reported purchasing a gun during the pandemic. This percentage is similar to recent reports that range from 6.5% (national sample of women and men) to 8% (national sample of men) (Hill et al., 2021, Tavernise, 2021). Most respondents were 35 years of age or older (65%). Over half of the respondents identified as female (52%). The sample included non-Hispanic whites (19%), non-Hispanic blacks (22%), Latinos (20%), Asians (36%), and respondents of other races and ethnicities (3%). Most respondents reported being born in the United States (76%), and, at the time of the interview, over two-thirds of respondents were living in the South or West (68%). Nearly half of respondents reported being married (46%) while the average respondent reported having less than one child in the home. Over one-third of respondents reported having a four-year college degree or higher (39%). The average annual household income was between $50,000 and $59,999. Median household income was also between $50,000 and $59,999. The interquartile range of household income was between $25,000 to $29,999 and $85,000 to $99,999. Most respondents reported being employed before and during the pandemic (70%). Although most respondents attended religious services rarely or never (66%), a large majority of respondents reported at least some degree of religiousness (81%). Finally, the sample included Republicans (24%), Democrats (59%), and respondents who reported being an independent or having no affiliation with a political party (17%).

### 3.2 Regression analyses

In Table 2, we regressed pandemic gun purchases on sociodemographic variables. The odds ratios (ORs) shown in this table can be manipulated ([OR − 1] × 100) to describe the percentage difference in the odds of having purchased a gun during the pandemic for each one-unit change in the independent variable of interest. Compared with respondents 60 years of age and older, the odds of purchasing a gun during the pandemic were 213% ([3.13 – 1] × 100) higher for respondents ages 18 to 24 (OR = 3.13, p < 0.001), 249% higher for respondents ages 25 to 44 (OR = 3.49, p < 0.001), and 138% higher for respondents ages 45 to 59 (OR = 2.38, p < 0.01). The odds of purchasing a gun during the pandemic were 96% higher for men (OR = 1.96, p < 0.01) and 307% higher for respondents who were born in the United States (OR = 4.07, p < 0.001). Compared with residents of the South, the odds of purchasing a gun during the pandemic were 48% lower for residents of the Northeast (OR = 0.52, p < 0.05). The odds of a pandemic gun purchase were comparable for residents living in the South and Midwest/West and across race and ethnicity (racial and ethnic minorities compared with non-Hispanic whites).

Model 2 added family variables to Model 1. The odds of a pandemic gun purchase did not vary by marital status or number of children in the home. The patterns reported in Model 1 were replicated in Model 2.

Model 3 introduced socioeconomic variables to Model 2. Relative to respondents with less than a high school degree, the odds of purchasing a gun during the pandemic were 64% lower for respondents with a graduate degree (OR = 0.36, p < 0.05). Compared with respondents who became unemployed during the pandemic, the odds of a gun purchase during the pandemic were 53% lower for respondents who were continuously unemployed before and during the pandemic (OR = 0.47, p < 0.05). The odds of a pandemic gun purchase did not vary by household income. For the most part, the patterns reported in Model 2 were replicated in Model 3. After controlling for socioeconomic variables, the age difference between 45 and 59 and 60 and older was attenuated to insignificance (OR = 2.07, p > 0.05).

Model 4 added religion variables to Model 3. Compared with respondents who identified as not at all religious, the odds of purchasing a gun during the pandemic were 190% higher for respondents who reported becoming *more* religious during the pandemic (OR = 2.90, p < 0.01) and 279% higher for respondents who reported becoming *less* religious during the pandemic (OR = 3.79, p < 0.001). The odds of a pandemic gun purchase did not vary by religious service attendance. The patterns reported in Model 3 were replicated in Model 4.

Finally, Model 5, the full model, introduced political party affiliation to Model 4. Compared with respondents who identified as Republicans, the odds of purchasing a gun during the pandemic were 68% lower for Democrats (OR = 0.32, p < 0.001) and 50% lower for independents and respondents who reported no affiliation (OR = 0.50, p < 0.01). Once again, the patterns reported in Model 4 were mostly replicated in Model 5.

## 4 Discussion

In this paper, we explored the social patterning of recent gun purchases to advance a contemporary social epidemiology of the pandemic arms race. Our key results suggest that pandemic gun purchasers tend to be male, younger, US-born, less educated, recently unemployed, experiencing changes in their religious beliefs, Republicans, and residents of southern states. Although our findings for sex, educational attainment, unemployment status, political affiliation, and region of residence are generally consistent with previous studies of pre-pandemic gun ownership, the null patterns for race/ethnicity, household income, marital status, and the presence of children are inconsistent with previous research (Azrael et al., 2017, Dowd-Arrow et al., 2019, Ellison, 1991, Ellison et al., 2021, Goss, 2017, Hepburn et al., 2007, Kelley and Ellison, 2021, Legault, 2008, Mencken and Froese, 2019, O’Connor and Lizotte, 1978, Parker et al., 2017, Smith et al., 2019, Vegter and Kelley, 2020). Our results for age, race, and southern residence are consistent with recent research on pandemic gun purchases, and our findings for education and employment are inconsistent with this work (Hill et al., 2021). To our knowledge, our examination of nativity status and religious change is unprecedented in the literature. In other words, we are among the first to formally document a new population of pandemic gun owners that is characterized by youth, US-nativity, and religious volatility.

Pandemic gun purchases and gun-related violence have been at least partially motivated by widespread uncertainty and fear, trauma and loss (Caputi et al., 2020, Kravitz-Wirtz et al., 2021, Lyons et al., 2021). One reason for these motivations is the idea that guns may somehow empower their owners by contributing to a subjective sense of personal control over life (Hill et al., 2020, Mencken and Froese, 2019). These themes clearly dovetail with our profile of recent gun purchasers. Some men may buy new guns or stockpile even more guns during the pandemic as a behavioral expression of strength, aggression, competition, or some other element of hegemonic masculinity (Pfaffendorf et al., 2021). While younger people typically engage in riskier lifestyles (Cockerham, 2005), they have been especially politically active during the pandemic. Now they are arming themselves. The nativity disparity in recent gun purchases may reflect laws against undocumented immigrants owning firearms. Differences by nativity status and party affiliation are also consistent with political conservatism and recent surges in nationalism and xenophobia (Perry et al., 2020). The recently unemployed may be arming themselves in response to the myriad threats to material security or the prospect of suicidal behavior (McCourt, 2021, Sutherland et al., 2021). Finally, our findings suggest that recent gun purchases may be associated with different forms of religious change. We found that becoming more *and* less religious during the pandemic may somehow inspire the acquisition of guns. The very concept of religious change may reflect coping with uncertainty or strain through an active search for meaning (becoming more religious) or an intra-psychic struggle with one’s religious beliefs (becoming less religious) (Ellison and Lee, 2010). Religious volatility during a pandemic could be indicative of underlying psychosocial strains (e.g., fear of infection, loss of family members, unemployment, and social isolation). These stains could conceivably motivate some Americans to purchase guns to enhance a sense of security.

Our study should be considered in the context of several limitations. Because our analyses are based on a cross-sectional design, no causal or temporal inferences can be made. Our measurement of gun purchases also lacks nuance. The ideal measurement would indicate first-time purchasers and previous owners. It would also be informative to assess precise motivations for purchases. Another potential limitation is that we were unable to consider neighborhood factors (e.g., community crime) that could also motivate gun purchases. Future research should explore how these factors impact and interact with the effects of individual-level correlates on pandemic gun purchases. Finally, we note that our data include an oversample of Asian Americans (by design) and, as a result, Democrats. While these unique sample characteristics could impact our descriptive statistics, we are not overly concerned. For example, we find that approximately 6.18% of US adults purchased a gun during the pandemic. Other studies of pandemic gun purchases have reported 6.5% (Tavernise, 2021). Our sample is also unlikely to bias our regression coefficients since we adjust for race and political identification (Winship and Radbill, 1994). We acknowledge that the veracity of our findings is contingent on replication in future research.

According to media accounts and recent reports, many pandemic gun purchases were made by first-time gun owners and people struggling with insecurity and fear (Tavernise, 2021). Our analyses underscore the need for public health initiatives designed to enhance gun-related safety during pandemics, including, for example, addressing underlying motivations for recent gun purchases and improving access to training programs. Guns are being purchased at record rates during periods of concurrent crises in public health, institutional decline, and social inequality. Gun-related violence is also on the rise. We need to better understand how guns are being marketed and why people feel the need to purchase guns for the first time during a pandemic. We need to assess the degree to which the pandemic arms race is comorbid with other issues related to public health (e.g., mental health) and public safety (e.g., crime). We need to begin to think about guns as fundamental elements of the pandemic and pandemic lifestyles in the US. Finally, we need to do a better job of communicating the risks associated with guns, especially during times of crisis.

These avenues for research and programmatic intervention are vital for at least two reasons. First, it is unclear whether, or to what extent, recent gun buyers—especially first-time buyers—are well-versed in gun safety, marksmanship, or laws regarding the legal issues surrounding owning and carrying weapons. The spike in gun sales and business closures during the pandemic are likely to have increased demand while limiting opportunities for training related to the safe handling of firearms. The “great ammunition famine,” including widespread shortages of and skyrocketing prices for many calibers of handgun ammunition (Fernandez, 2021), may also constrain new gun buyers from regular target practice at shooting ranges, which is commonplace for many safe and experienced gun owners.

Second, the pandemic arms race is occurring in a broader dystopian context. Nearly 700,000 Americans have died from COVID-19 during a global pandemic (Coronavirus Resource Center, 2021). The U.N. Secretary-General described a recent report from the Intergovernmental Panel on Climate Change as a “code red for humanity” (Rice, 2021). The unequal distribution of income in the US is approaching levels recorded during the Gilded Age (Wolff-Mann, 2021). Homeless shelters are preparing for a looming eviction crisis (Brookbank, 2021). Homicides are spiking (Rosenfeld and Lopez, 2020). Americans are deeply concerned about crime in their communities (McCarthy, 2020). National political protest and unrest have been widespread across the ideological spectrum, stemming from left-wing concerns about fascism (e.g., Antifa) and police violence (e.g., the murder of George Floyd) and right-wing anxieties about the federal government (e.g., Boogaloo Boys), election fraud (e.g., the U.S. Capitol riot), and demographic change (e.g., Proud Boys). Lastly, we find ourselves living in a veritable panopticon where corporations and governments regularly observe us without our knowledge or consent (Burns, 2021). All of this is to say that the toxic stew of guns mixed with pervasive uncertainty, dysphoria, and despair could conceivably contribute to lethal acts of anger (e.g., road rage) and violence (e.g., mass shootings). Future research is strongly encouraged to monitor these possibilities.

### CRediT authorship contribution statement

**Terrence D. Hill:** Conceptualization, Methodology, Writing – original draft, Writing – review & editing. **Ming Wen:** Conceptualization, Methodology, Formal analysis, Visualization. **Christopher G. Ellison:** Writing – original draft, Writing – review & editing. **Guangzhen Wu:** Writing – original draft, Writing – review & editing. **Benjamin Dowd-Arrow:** Writing – original draft, Writing – review & editing. **Dejun Su:** Funding acquisition, Investigation, Writing – original draft, Writing – review & editing.

## Declaration of Competing Interest

The authors declare that they have no known competing financial interests or personal relationships that could have appeared to influence the work reported in this paper.
